# Daughter Preference and Contraceptive-use in Matrilineal Tribal Societies in Meghalaya, India

**DOI:** 10.3329/jhpn.v31i2.16393

**Published:** 2013-06

**Authors:** Pralip Kumar Narzary, Shilpi Mishra Sharma

**Affiliations:** ^1^Post-Graduate Department of Population Studies, Fakir Mohan University, Nuapadhi, Balasore 756 020, Odisha, India;; ^2^Institute of Health Management Research, 1 Prabhu Dayal Marg, Sanganer Airport, Jaipur 320011, Rajasthan, India

**Keywords:** Contraceptives, Daughter preference, Matriliny, India

## Abstract

Although son preference in patrilineal society is an established fact, daughter preference in matrilineal society is not thoroughly examined. Very few studies have been carried out on the issue. This paper attempts to explore the daughter preference and contraceptive-use in matrilineal tribal societies in Meghalaya, India. Data from the National Family Health Survey 1998-1999 have been used in this study because, among the large-scale surveys, only this dataset allows identification of matrilineal sample. Mean, percentage, and standard deviation are computed in the present study. Further, the data have been cross-tabulated, and logistic regression has been run through SPSS (version 15). Among the ever-married matrilineal women, 17% desired more sons than daughters but 18.2% desired more daughters than sons. About 11% of ever-married women could achieve their desired sex composition of children. However, a very striking finding suggests that, even after achieving desired sex composition of children, as high as 61.8% of women were still not using contraception mainly because of programme factors while one-fourth were still depending on temporary methods. The rest 13.2% adopted terminal method of contraception, which calls for immediate attention of planners. With the increase in the number of sons but without daughter, contraceptive-use drastically decreased. The most desired sex composition of children seems to be two daughters and a son. Absence of daughter with increase in the total number of sons increased the desire for additional children. Every woman with two or more sons but without daughter wanted the next child to be a daughter. Thus, there are ample evidences to draw the conclusion that there is, in fact, a daughter preference in the matrilineal tribal societies in Meghalaya, India. Policy-makers may, thus, target the women who have achieved fertility and should ensure that daughter preference does not lead to the negligence to sons.

## INTRODUCTION

It is amazing that ethnic, linguistic and religious groups co-exist, along with their own varied cultural systems in India. Matriliny, one of the peculiar social systems has almost disappeared in South India (1) but is still cherished in the state of Meghalaya. Although true matrilineal society does not exist anywhere in the world today, it is commonly agreed that three basic elements of matriliny exist in the present-day matrilineal societies, viz. descent through mother (family name through mother), matrilocal residence system (husband lives in wife's residence), and inheritance of parental property by daughter. Any society where these characteristics exist is considered to be matrilineal. All of these three characteristics are strongly prevalent among the Khasi, Garo, and Jaintia tribes in Meghalaya, qualifying to be matrilineal societies.

Matrilineal society exists in various forms among the tribes of African countries, Maldives, in some parts of South-East Asian countries, and among a few communities in India. Among them, Minangkabau of West Sumatra is regarded as the largest matrilineal society in the world (cited in Tanius 1983). In India, matrilineal social system is still followed in small pockets of Kerala, Laksha Dweep, and Meghalaya. Meghalaya is a small state located in the northeastern part of India. The total population of the state according to 2011 Census is 29,64,007, of whom about 86% is tribal, and about 70% of them follow Christian religion. It is a state with the largest proportion of population following matrilineal system. In Meghalaya, the contraceptive-use of 20.2% was the lowest, and the total fertility rate of 4.57 was the highest in the country (2) during the study period. By 2005-2006, contraceptive prevalence rate has increased to 24.3% but is still the lowest in the country, and the total fertility rate, which has declined to 3.80, is the third highest (next to Bihar and Uttar Pradesh) in the country (3). Hence, there is a need to examine linkage between daughter preference and contraceptive-use, especially to know whether the matrilineal tribal women/couples terminate the childbearing process once they achieve the desired number of children and sex composition of their children. This becomes necessary for bringing down the fertility level and improving reproductive health.

Son preference in patrilineal society, especially in India, is a well-established fact. Some studies even showed female foeticide and infanticide in few states of the country due to son preference. The declining sex ratio (decline in the number of females compared to males) in the country is also the testimony to a strong son preference. Based on the Provisional Census Data 2001, a study has classified a few districts of the country on the basis of 0-6 years’ sex ratio to the extent of DEMARU, meaning “daughter eliminating male aspiring rage for ultrasound” (4). However, sex preference in a matrilineal society does not seem to be well-examined. So, very little is known about sex preference of children in the matrilineal tribal societies, especially in India.

The birth of a female child in Jaintia society in Meghalaya is hailed with a great joy. It may not be an exaggeration to say that parents often feel happier to have a female child for the simple reason of being sure of the continuity of the family and the clan (5). However, there seems to be no discrimination in the upbringing of the male and female children (5,6). In Jaintia society, girls get proper attention of the parents regarding education and health (7). Similarly, a study claims that, in matrilineal societies of Meghalaya, especially female children are considered as assets and thereby get better treatment. Girls are the progenitors of the family and of the clan because children follow the mother's lineage but not that of the father (8). Further, a strong daughter preference is seen among the Khasi matrilineal tribal women in Meghalaya (9). So, it can be said that there is a preference for daughter in matrilineal tribal societies in Meghalaya. However, in a study conducted in West Sumatra, it was found that, in matrilineal Minangkabau society, there were more respondents who wished for “more boys than girls” among the four regions, except in the rural area of Matriarchat Centre (10). It, thus, looks apparent that fertility preference in the matrilineal societies around the world is not conclusive.

The present study hypothesized that women/couples who have had their ideal number of children with desired sex composition are supposed to practise terminal methods of contraception.

## MATERIALS AND METHODS

Among the various large-scale demographic surveys, only National Family Health Survey 1998-1999 (NFHS-2) provides information on names of the tribes, by which one can identify a woman whether she follows matrilineal culture or not. Even the recent NFHS 2005-2006 (NFHS-3) data do not provide this information. On the other hand, surveys conducted by individual(s) do not provide access to their data for in-depth analysis by others. As a result, the NFHS-2 data have been used for the present study (2,11). Lack of accessibility to the datasets used by only a few available earlier studies (12,13) based on single tribe, small area, and purposively selected small sample-size necessitated the use of this dataset.

The NFHS-2 covered women from various tribes and communities but only matrilineal tribal women—Khasi, Garo, and Jaintia—have been selected for the present study. These are the only three matrilineal tribes in the state of Meghalaya; 786 (83.17%) out of 945 ever-married women were identified as belonging to any of the matrilineal tribes, of whom 380 were from Khasi, 299 from Garo, and 107 from Jaintia tribe. Of the 786 matrilineal respondents, 669 (85.1%) were found married during the study period whereas the remaining 117 (14.9%) were formerly married.

While dealing with the ideal number of children, women who gave non-numeric responses have been dropped from the analysis; so, the total sample comprised 716 ever-married women. Initially, the status of contraceptive-use was examined only among the currently-married women who have achieved their ideal number of children with desired sex composition. Hence, the sample comprised only 68 currently-married women, although the reasons for non-use of contraceptives were assessed only among the non-users (42 currently-married women). Later, the contraceptive-use was examined by number of living daughters and sons among all the currently-married women (669 cases). To assess the desire for additional children, women being sterilized or infecund (96 cases) were removed from the analysis. So, the analysis was based on only 573 currently-married women who were capable of reproducing. To assess the sex preference of the next child, only the currently-married women who wanted more child(ren) and reported to have either daughter or son were retained, which led to the final analysis of 222 cases. On the other hand, 170 currently-married women who said “it is up to God” or “does not matter” were removed from the analysis because these answers do not allow the assessment regarding sex preference. Most of the analysis excluded the formerly-married women because normally they are not exposed to the risk of pregnancy in the Indian social context.

To fulfill the objectives of the study and to test the set hypothesis, mean, percentage, and standard deviation were computed. Further, data have been cross–tabulated, and logistic regression has been run to find out possible relationship between the variables, using SPSS (version 15).

## RESULTS

### Fertility behaviour and child loss

In the present study, fertility has been measured by ‘children ever born’ while child loss by ‘number of children who died’. Table 1 shows that average number of children ever born to the ever-married women in the matrilineal tribal societies were 3.70, with a very high standard deviation of 2.57 and slightly more number of sons ever born than the daughters. Mean number of living children was found to be 3.19, with a slightly higher number of living sons (1.65) than daughters (1.53). Standard deviation of the number of living children came out to be very high (2.17) compared to both number of living sons and daughters when computed separately. Mean number of children dying among the matrilineal women in Meghalaya was 0.51, with a standard deviation of 1.02. Mean number of sons (0.28) and daughters (0.24) who died is almost equal. This indirectly indicates the equal treatment to children in the community; of course, this might be during infancy and/or childhood.

It has been found that mean ideal number of children for ever-married women in matrilineal tribal society is 4.95, with a very high standard deviation of 2.05 (Table 1). It should be noted that 8.9% (70 cases) of women did not give any numeric response to this question (results not shown). The mean ideal number of sons (2.40) and daughters (2.39) shows no such differences, although the standard deviation is slightly higher for sons (1.24) than that of daughters (1.16). However, it should be noted that difference in mean value of ideal number of sons and daughters alone cannot ascertain if there is any sex preference of children because this is highly affected by the extreme values and, on the other hand, by the number of living children itself. One of the reasons for very high ideal number of children and high fertility rate in the state may be the very high mortality of infants and under-five children (IMR 89—highest in the country and under-five mortality 122—the third highest in the country) (2). However, a study noted that the local tribes in Meghalaya were/are pro-natalists, and they proudly proclaim to be so. Population increase is considered a boon, and the children are considered gifts of God. The study further states that the people believe: as long as they are able to feed the children, the number is not a problem (8). Some studies (8,12) suggest reasons for high fertility simply as fear of psychosis against voluminous immigration from neighbouring countries and non-tribals from other parts of the country. This may change the demographic structure of the state, and the prestige and status attached to large family-size in the society (8,12).

**Table 1.** Mean number and standard deviation of children ever born, number of living children, ideal number of children, and number of children who died

Variable	Mean/Average	Standard deviation
Children ever born	3.70	2.57
Son ever born	1.93	1.77
Daughter ever born	1.65	1.52
Number of living children	3.19	2.17
Number of living sons	1.65	1.42
Number of living daughters	1.53	1.34
Ideal number of children	4.95	2.05
Ideal number of sons	2.40	2.39
Ideal number of daughters	1.24	1.16
Number of children who died	0.51	1.02
Number of sons who died	0.28	0.67
Number of daughters who died	0.24	0.57

Total cases=786

### Ideal sex composition of children

**Figure 1.**
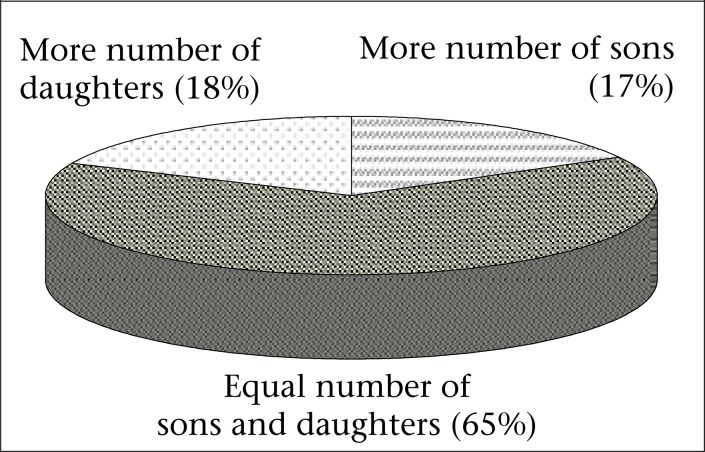
Ideal number of children

Conventional way of looking at the sex preference for children is to find out the ideal sex composition of children or, in other words, the difference in ideal number of sons and daughters, which is attempted here too. It has been found that, for about 65% of ever-married women, the ideal number of daughters and sons is equal whereas 17% of the women have reported to desire more sons than daughters but 18.2% of the women have reported to desire more daughters than sons (Figure 1). In other words, the Daughter Preference Index (DPI) is 1.07. This result shows slight preference for daughters over the sons. However, as most of the societies pass through a transitional phase due to several external and internal forces and matrilineal societies of Meghalaya are no exception (8,13-15), the importance of daughter is losing its significance; so, preference for daughter is less apparent. Elsewhere also, changes in matrilineal cultural traits are partly or completely reported, particularly among the Garos of Assam (16). Further, son preference is weak in populations with both high and low fertility (17).

### Differences between living and ideal number of children

**Figure 2.**
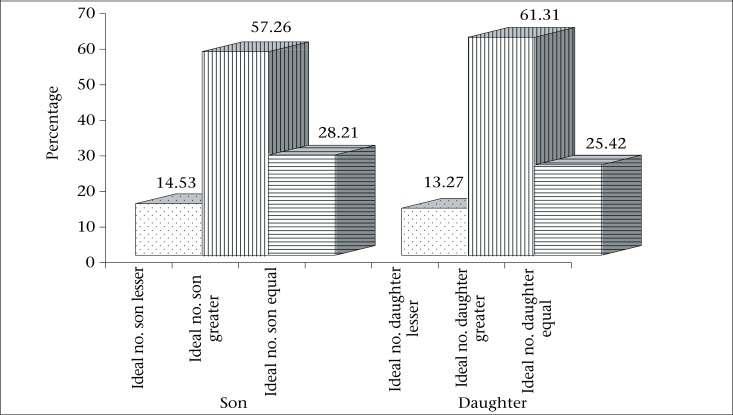
Difference between numbers of ideal and living children

It is generally argued that the ideal number of children is greatly affected by the number of living children; so, it will be of worth to check this relationship. Figure 2 depicts that, for about 61.3% of ever-married women, the ideal number of daughters is higher than the number of living daughters while 13.3% of the women have higher number of living daughters than the ideal number. For about one-fourth of the women, it is equal. Thus, it can be said that the ideal number of daughters is not affected by the number of living daughters in matrilineal tribal society.

It has been found that, for about 57.3% of the women, the ideal number of sons is greater than their living sons. Interestingly, only 14.5% of the women have more number of living sons than the ideal number of sons. However, slightly more than one-fourth of the women have equal number of living and ideal number of sons (Figure 2). This result also shows a similar pattern in terms of difference between ideal number of daughters and number of living daughters. The differences between living and ideal number of sons and between living and ideal number of daughters also ascertain the existence of daughter preference in the matrilineal tribal societies in Meghalaya.

### Factors affecting ideal sex composition of children

It is imperative to know which groups of women prefer more number of daughters than sons, and an attempt is made here to address this question. The result in Table 2 shows that daughter preference is comparatively more apparent among women in 25-34 years age-group, non-literate, highly-educated, formerly-married, and non-working women as well as women from male-headed households, urban households with low standard of living, and among Jaintia and Garo tribes.

Except for non-literate women, preference for more daughters steadily increased with increase in the level of education. Daughter preference is the highest among the non-literate and women with the highest level of education. Almost a similar pattern is observed in the case of educational level of husband as well. However, study on son preference exhibits significant negative relationship between husband's education and son preference, although women's education has no significant relationship (18). This connotes that education has not much to do with the sex preference of children, or because educated Khasi women are culturally more sensitive and as such found to be strictly following the cultural taboos and norms (9).

Preference for daughters was more pronounced among the formerly-married women than the currently-married ones. It may be because they felt that it was ideal to have more daughters for old-age security. Similarly, women who were currently not working might not be economically strong and, hence, they looked forward to daughters for economic support during their old age, thereby preferring more daughters. On the contrary, as working women were economically independent, the percentage of women who preferred more sons was higher among them. Another interesting point to note is that, although percentage of rural and urban women who desired to have more daughters was almost equal, the percentage of rural women who desired to have more sons was about 8 percentage-points higher than urban women. Preference for daughter declined quite apparently with the increase in the standards of living. A similar pattern was observed even for sons, which means that sex preference (for either) declined with the increase in the standards of living. Another study on son preference also showed similar relationship (18). Daughter preference is more perceptible among the Jaintia and Garo women than the Khasi whereas percentage of women who preferred son is almost equal across all the three groups of women. Daughter preference was less among the Khasi because they have more contact with other patriarchal communities for the state capital being located in Khasi Hills where the transition is passing at a much faster pace.

### Achieved fertility and contraceptive-use

In the present paper, ‘achieved fertility’ has been defined as equal number of living and ideal number of daughters and equal number of living and ideal number of sons. In other words, sex composition of living children remains the same with sex composition of ideal number of children. It has been found that almost 89% of ever-married women could not have achieved fertility or could not achieve their desired sex composition of children but 11% (77cases) of women could achieve their desired sex composition of children (Table 3). It is assumed that later group of women should not desire to have additional child and rather take recourse to contraception, mostly a terminal method.

Result on contraceptive-use (Table 4) clearly indicates that, among the women who achieved fertility and were currently-married, 61.8% (42 cases) were still not using any method of contraception. On the other hand, 25% (17 cases) were still depending on temporary methods of contraception, and only 13.2% (9 cases) adopted terminal method of contraception. Similarly, the field survey conducted in 1999 among 400 Khasi women found that more than 70% of the current contraceptive-users were relying on temporary methods (12). Thus, the hypothesis “women/couples who have had their ideal number of children with desired sex composition are supposed to practise terminal methods of contraception” may be rejected.

However, it is essential to know as to why such small percentages of women adopted contraception, especially a terminal method.

### Reasons for not using contraception

Among the women who had ‘achieved fertility’, the reasons cited for not using any method of contraception reflect that majority of women (38%) did not use mainly because of programme factors (knew no method, knew no source, lack of access or too much cost); slightly more than one-fourth (26.2%) were not using because of individual factors (wanted more children, were pregnant, husband opposed, health concern, or side-effects); and the remaining (35.7%) were not using for other reasons (Table 5). However, a study conducted among the Khasi women in East Khasi Hills district found that the main reasons for never using any method of contraception pertaineed to the client factors (45.8%), followed by cultural factors (32.9%), health factors (14.2%), and programme factors (7.1%). The study included ‘difficult to get method’ and ‘can't afford’ as programme factors; ‘side-effects’ and ‘difficult to get pregnant’ as health factors; ‘against religion’ and ‘cultural taboos’ as cultural factors, and ‘lack of knowledge’, ‘husband opposed’, ‘not necessary’, ‘felt scared’, ‘wanted more child’, ‘wanted a daughter’ and ‘husband away’ as client factors (14). Thus, in the study, if ‘lack of knowledge’ and ‘felt scared’ were clubbed into programme factors, the similar result might have come out.

**Table 2.** Differences in ideal sex composition of children by background characteristics

Background characteristics	Equal^*^	More daughters^**^	More sons^***^	n
Age-group (years)				
Below 25	66.1	18.7	15.2	171
25–34	60.8	19.4	19.7	309
Above 34	69.1	16.1	14.8	236
Level of education (women)				
No education	63.8	21.3	14.9	235
Literate, <middle school complete	64.1	14.9	21.0	309
Middle school complete	70.4	18.4	11.2	98
High school complete and above	63.7	21.6	14.9	74
Marital status				
Currently married	65.6	17.4	16.9	608
Formerly married	60.2	22.2	17.6	108
Working status				
Currently not working	65.5	18.5	15.9	383
Currently working	64.0	17.7	18.3	333
Level of education (husband)				
No education	62.0	20.3	17.7	192
Literate, <middle school complete	68.0	17.3	14.7	278
Middle school complete	62.0	14.0	24.0	121
High school complete and above	65.0	20.8	14.2	120
Sex of the head of household				
Male	64.3	18.7	17.0	566
Female	66.7	16.0	17.3	150
Place of residence				
Urban	69.9	19.6	10.5	143
Rural	63.5	17.8	18.7	573
Household standard of living				
Low	60.5	20.7	18.8	309
Medium	67.1	16.9	16.0	343
High	76.9	9.6	13.5	52
Religion				
Christian	63.9	19.0	18.0	593
Others	69.1	18.7	12.2	123
Tribe				
Khasi	67.5	15.5	17.0	329
Jaintia	61.9	21.0	17.1	105
Garo	62.8	20.2	17.0	282
Total percentage	64.8	18.2	17.0	100.0
Total cases	464	130	122	716

Some of the figures may not add up to total due to missing cases/system;

^*^Ideal number of daughters and sons is equal;

^**^Ideal number of daughters is greater than that of sons;.

^***^Ideal number of sons is greater than that of daughters

**Table 3.** Achieved fertility

Fertility status	Frequency	Percentage
Not achieved	639	77
Achieved	89.2	10.8
Total	716	100.0

**Table 4.** Contraceptive-use

Contraceptive-use	Frequency	Percentage
Not using any	42	61.8
Pill	5	7.4
IUD	5	7.4
Condom	3	4.4
Female sterilization	9	13.2
Periodic abstinence	3	4.4
Others	1	1.5
Total	68	100

**Table 5.** Reasons for not using any method of contraception

Reason for not using any method of contraception	Frequency	Percentage
Personal factor	11	26.2
Programme factor	16	38.1
Others	15	35.7
Total	42	100.0

Personal factor=Menopause, hysterectomy, wanted more children, were pregnant, husband opposed, health concern, and side-effects. Programme factor=Knew no method, knew no source, lack of access, and too much cost. Others=Missing and reasons not cited

In the present study, some women wanted to have more children even after attaining desired sex composition of children; this might be due to the very high mortality of infants and under-five children (although it is not reflected in the cited reasons) because they were not sure whether their children would survive. The reasons given for not using any method of contraception showed that the women who had ‘achieved fertility’ could be motivated to use contraception through improvement in the reproductive and child health programmes.

### Number of living daughters and contraceptive-use

**Figure 3.**
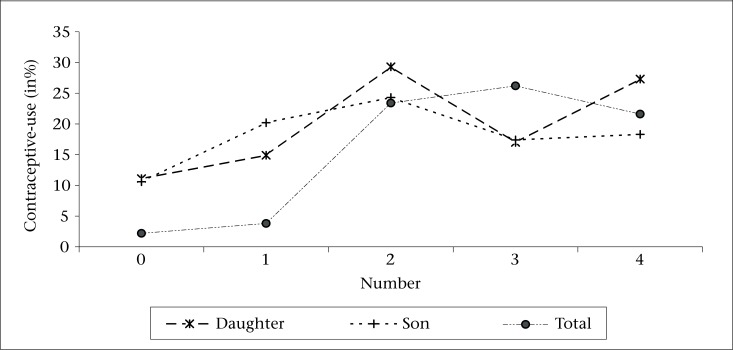
Current contraceptive-use by number of children

It is an established fact that in patriarchal societies, the number of living sons is one of the most important determining factors of contraceptive-use. On the contrary, a strong preference for girls was a significant determinant of contraceptive-use among the matrilineal Garo women in Bangladesh (19). Here also, an attempt is made to assess the contraceptive-use among the currently-married women by the number of both living daughters and sons. Result in Figure 3 shows that percentage of women using contraception increased up to three living children, implying preference for three children, irrespective of sex composition. It is apparent that contraceptive-use increased up to two living daughters as well as sons, although the level of use was comparatively higher when they had two daughters and thereafter became erratic. It implies that the relationship between number of living daughters/sons and contraceptive-use does not seem to be very straightforward. It is, therefore, difficult to draw a strong conclusion from this bivariate relationship. However, the better way of looking at this relationship may be to control any one of the three independent components (Table 6).

One of the very interesting findings is that when currently-married women had two living sons with no daughter, about 27% used contraception. However, when they had three sons without any daughter, only 20% used contraception. Similarly, when they had four sons without any daughter, no women used contraception (Table 6). These results strongly indicate the growing desire of women to beget a daughter with increase in the number of living children.

Result indicates that, among different sex composition of children, the highest percentage of women using contraception was observed when they had two daughters, or two daughters and a son. On the contrary, a study on son preference showed the highest contraceptive-use, with two sons and a daughter (20). So, the present study finds that couples/women in matrilineal societies do not want only daughters, they also want to have a son. Further, it also signifies that the best sex composition of children for matrilineal couples/women in Meghalaya was at least two daughters and a son, thereby indicating the prevailing daughter preference. However, this result is slightly in contrast to the ideal number of daughters and sons because, while answering about ideal number of children, they seemed to relate the same to total number of living children.

### Desire for additional child(ren)

A way of looking at daughter preference is to examine the desire for additional child(ren) by total number of living daughters and sons (Table 7). The percentage of women desiring to have additional child(ren) was the highest when they had only a son and no daughter. This was even higher than among women without any child. This may be because once the women had given birth to a child, they were sure of their fecundity and, hence, wished to have an additional child. When both children were daughters, about 67% of the women wanted an additional child. In other cases, if they did not have any daughter, as high as 77% of the women wanted an additional child. Similarly, when they had three children, and all of them were daughters, about 67% of the women desired an additional child but when all were sons, about 69% of the women wanted more child(ren). Further, when they had four children without any son, slightly less than half of the women desired additional child(ren) but when they did not have any daughter, as high as 83% wanted an additional child. One can infer from the above that this pattern was due to the daughter preference. However, to confirm this proposition, attempt was made to see their desire for sex of the next child.

**Table 6.** Percentage of currently-married women using contraception by total living daughters and sons

Daughter	Son	Contraceptive-use	n
0	0	2.2	45
	1	7.4	54
	2	27.3	33
	3	20.0	15
	4	0.0	6
	Total	11.1	153
1	0	0.0	51
	1	20.5	73
	2	22.6	53
	3	12.5	16
	4	13.6	22
	Total	14.9	215
2	0	27.3	22
	1	32.6	46
	2	25.6	39
	3	19.2	26
	4	50.0	14
	Total	29.3	147
3	0	25.0	8
	1	15.0	20
	2	21.4	28
	3	23.5	17
	4	0.0	15
	Total	17.0	88
4	0	40.0	15
	1	60.0	5
	2	25.0	20
	3	8.3	12
	4	21.4	14
	Total	27.3	66
Total		18.7	669
Total		18.7	669

‘Total’ in the bottom of rows (except the last) refers to the number of currently-married women with various combinations of daughters and sons; 'total’ in the last row indicates total number of currently-married women

**Table 7.** Percentage of currently-married women desiring additional child(ren) by total number of living daughters and sons

Daughter	Son	Want more child(ren)	n
0	0	91.2	34
	1	98.0	50
	2	76.7	30
	3	69.2	13
	4	83.3	6
	Total	88.0	133
1	0	94.1	51
	1	79.7	64
	2	69.6	46
	3	53.3	15
	4	72.2	18
	Total	78.4	194
2	0	66.7	21
	1	50.0	38
	2	41.9	31
	3	54.5	22
	4	36.4	11
	Total	50.4	123
3	0	66.7	6
	1	57.9	19
	2	50.0	22
	3	33.3	12
	4	54.5	11
	Total	51.4	70
4	0	50.0	14
	1	50.0	2
	2	40.0	15
	3	45.5	11
	4	54.5	11
	Total	47.2	53
Total		68.4	573
Total		68.4	573

‘Total’ in the bottom of rows (except the last) refers to the number of currently-married women with various combinations of daughters and sons; 'total’ in the last row indicates the number of currently-married women who were fertile (infecund and women who adopted sterilization are excluded)

### Desire for additional daughter(s)

Table 8 presents the result of sex preference of the next child (here two categories of responses “sex does not matter” and “it is up to God” have been excluded from the analysis). The result shows that, among the women who wanted additional child(ren), 60.4% wanted a daughter, thus yielding an overall daughter preference index of 1.5. Further, it has been found that half of the women desired their first child to be a daughter whereas the remaining half desired their first child to be a son. However, when the women had one living child and it was a daughter, their desire for the next child to be a girl was very low (13%). On the contrary, if it was a son, 94% of the women wanted the next child to be a girl. From two sons onward, when women did not have any daughter, everyone wanted the next child to be a girl. It sufficiently implies that the desire for a daughter was much more pronounced among the women of higher parity, who did not have a daughter. On the contrary, when all the four living children were daughters, no woman desired the next child to be a girl. This signifies that, although there is daughter preference in the matrilineal societies, there is no negligence to male children.

**Table 8.** Percentage of currently-married women preferring the next child to be female by total number of living daughters and sons

Daughter	Son	Prefer next child to be female	n
0	0	50.0	14
	1	94.3	35
	2	100.0	20
	3	100.0	7
	4	100.0	5
	Total	88.9	81
1	0	13.3	30
	1	64.0	25
	2	95.0	20
	3	100.0	5
	4	100.0	9
	Total	59.6	89
2	0	0.0	9
	1	15.4	13
	2	50.0	4
	3	66.7	3
	4	0.0	1
	Total	20.0	30
		0.0	
3	0		1
		20.0	
	1		5
		20.0	
	2		5
		0.0	
	3		1
		50.0	
	4		2
		21.4	
	Total		14
4	0	0.0	6
	1	-	-
	2	0.0	2
	3	-	-
	4	-	-
	Total	0.0	8
Total		60.4	222
Total		60.4	222

‘Total’ in the bottom of rows (except the last) refers to the number of currently-married women with various combinations of daughters and sons; ‘Total’ in the last row indicates the number of currently-married women who wanted additional child(ren) and specified the desired sex

### Factors affecting desire for sex of the next child

As multivariate results can present more appropriate and accurate findings regarding the issue under investigation (21), we applied binary logistic regression (Table 9) to find out the factors affecting the desire for sex of the next child. Result shows that, after controlling for various socioeconomic factors, the desire for the next child to be a girl depended mainly on the sex composition of living children and standards of living. It is found that, compared to ‘others’ group (women who have both son and daughter or none), odds of desire to beget a female offspring is very strong (16.696) among the women who did not have any daughter. On the contrary, women without any son or with daughters only were less likely to desire the next child to be a girl (odds ratio 0.018). Compared to households with low standards of living, women from households with medium standards of living were less likely to desire the next child to be a girl. It was also true for women from households with high standard of living compared to those with low standard of living but the result was not that much significant. Except these two variables, none of the others was found to be statistically significant. Thus, the result clearly signifies that there was daughter preference in the matrilineal tribal societies in India. A study (18) also shows that sex composition of the living children has prominent impact on the desire for sex of the next child.

## DISCUSSION

This study confirms that, in contrast to son preference in patrilineal societies in general, there is a daughter preference in matrilineal tribal societies of Meghalaya, India. Thus, the question of whether ‘there is daughter preference in matrilineal tribal societies’ seems to be answered in this paper. In these societies, both attitudes and behaviour are significantly influenced by a long-standing preference for daughters that is deeply rooted in the matrilineal culture and traditions. However, even after reaching the desired fertility level or even after achieving their desired sex composition of children, majority of the women/couples do not use contraception in the matrilineal tribal societies. This rejects the hypothesis “women/couples who have achieved their ideal number of children with desired sex composition will use terminal methods of contraception.” Whether to have at all, how many, or what sex composition of children is the prerogative of the couple. Hence, whether to use contraception or not is also the concern of women/couples. However, planners and policy-makers can motivate the women/couples who have achieved their ideal sex composition of children to use contraception. Among the study women, the cited reasons for not using contraception mainly belong to programme factors. Thus, there is a need to improve the existing reproductive and child health programmes in the state. Also, the highest percentage of contraceptive-use was observed when women had two daughters and a son, indicating that it was the most desired sex composition for matrilineal tribal women in Meghalaya. Again, this implies that there is some evidence of a desire to have one son, if not more. However, from two children onward, when women did not have any daughter, everyone wanted the next child to be a girl. This shows a very strong desire to have at least a daughter in the family. Multivariate result indicates that the desire for the next child to be a girl depended mainly on the sex composition of living children and the standards of living. Women who did not have any daughter strongly desired the next child to be a girl while women having daughter(s) only were less likely to desire the next child to be a girl. The above facts suggest that there are ample evidences to draw the conclusion that there is daughter preference in the matrilineal tribal societies in Meghalaya, which may be the part and parcel of matrilineal culture itself. As efforts cannot change a culture easily which is not desirable as well, policy-makers should ensure that daughter preference does not lead to the negligence to sons.

### Limitations

As this survey did not consider the matrilineal aspect in the sampling design, the data so collected may not be fully representative of the matrilineal society. Thus, to probe deeply into the existence of daughter preference in the matrilineal tribal societies, specific survey is needed. Due to the limitation of the sample-size, tribe-wise results could not be presented. The results also seem to be affected by the very high non-numeric responses to the question on the ideal number of children, which should be noted in future studies because the non-numeric response to such an important question further reduces the sample-size and as such adversely affects the entire analysis.

**Table 9.** Determinants of sex preference of the desired additional child: Result of logistic regression [Dependant variable: Sex of desired additional child (male=0; female=1)]

Independent variable	Beta	Level of significance	Exp. Beta
Age (years)			
Below 25^R^			
25-34	-0.168	0.739	0.846
>34	-0.553	0.384	0.575
Educational level of women			
Non-literate^R^			
Literate, <middle school complete	0.117	0.805	1.124
Middle school complete	1.110	0.156	3.033
High school complete and above	-1.180	0.214	0.307
Husband's educational level
Non-literate^R^			
Literate, <middle school complete	0.157	0.773	1.170
Middle school complete	0.537	0.474	1.711
High school complete and above	1.339	0.097	3.815
Sex composition of living children			
Others^R^			
No daughter	2.815	0.000	16.696
No Sons	-4.031	0.000	0.018
Sex of head of the household			
Male^R^			
Female	-1.058	0.113	0.347
Standard of living			
Low^R^			
Medium	-1.128	0.013	0.324
High	-0.400	0.740	0.670
Place of residence			
Urban^R^			
Rural	-0.449	0.528	0.639
Name of the tribe			
Khasi^R^			
Garo	-0.183	0.546	0.833
Jaintia	-0.346	0.702	0.707
Religion			
Christian^R^			
Others	0.762	0.183	2.143
Constant	1.303	0.165	3.682

^R^Reference category

### Conclusions

Studies relating to matrilineal culture, especially on the tribes studied, are very limited and, hence, we had to rely on relatively old studies. Despite the limitations, the present study throws some light on the pattern of an existing social phenomenon—daughter preference—in the matrilineal societies of Meghalaya, India.
